# Supplementary material for: Properties and dynamics of meron topological spin textures in the two-dimensional magnet CrCl3
Source: Nat Commun. 2021 Jan 8;12:185. doi: 10.1038/s41467-020-20497-2 (PMC7794561; doi:10.1038/s41467-020-20497-2)
Supplement: Supplementary file 1 — Supplementary Information [file 41467_2020_20497_MOESM1_ESM.pdf]

Supplementary Information: Properties and dynamics of meron topological spin textures in the two-dimensional magnet $CrCl_3$

Mathias Augustin[1], Sarah Jenkins[2], Richard F. L. Evans[2], Kostya S. Novoselov[3,4] & Elton J. G. Santos[5,†]

[1] *School of Mathematics and Physics, Queen's University Belfast, BT7 1NN, UK*

[2] *Department of Physics, The University of York, York, YO10 5DD, UK*

[3] *Department of Material Science & Engineering, National University of Singapore, Block EA, 9 Engineering Drive 1, 117575, Singapore*

[4] *Chongqing 2D Materials Institute, Liangjiang New Area, Chongqing 400714, China*

[5] *Institute for Condensed Matter Physics and Complex Systems, School of Physics and Astronomy, The University of Edinburgh, EH9 3FD, UK.*

[†] *Corresponding author: esantos@ed.ac.uk*

Contents

1 Computational details **3**

2 Simulation movies **4**

3 Calculation of the meron and anti-meron radius **6**

4 Computing the topological quantum number **10**

5 Spin dynamics on other Cr-trihalide materials: CrX_3 (X=F, Br, I) **13**

1 Computational details

Ab initio methods: First-principles calculations were performed using the projector-augmented wave (PAW) method in the framework of density-functional theory (DFT), as it implemented in the VASP code [1]. The lattice parameters were optimized with a variable cell-shape relaxation method to minimize forces and internal pressure. The electron-ion interaction was described by means of PBE functional[2], where a plane wave kinetic energy cutoff was set to 500 eV. The tetrahedron method with Blöchl corrections and Γ-centered k-points meshes $15 \times 15 \times 1$ were used for integration in the irreducible Brillouin zone. This ensures a convergence of total energy to less than 10^{-6} eV/atom. We have taken into account strong correlation corrections in the frame of GGA+U simplified scheme introduced by Dudarev et al. [3]. We used an effective Hubbard $U_{eff} = U - J$ which values were previously obtained using linear response method[4] for $CrCl_3$ of 2.63 eV.

Atomistic approximations: Curie temperature calculations based on DFT simulations were mapped onto spin Hamiltonians by calculating total energies of different spin configurations in the 2×2 supercell as described in Ref.[5]. The magnetization as a function of temperature were calculated by using Monte Carlo method in the frame of the developed version of the Vampire atomistic spin dynamics package [6]. A $288\times166\times1$ model super-cell (191232 sites in total) based on the rectangular unit cell of honeycomb lattice (4 sites) with periodic boundary conditions was used.

The spin dynamics calculations are performed using atomistic spin dynamics within the stochastic Landau-Lifshitz-Gilbert equation[7] integrated with the Heun numerical scheme and 1 fs timestep [6]. Equilibrium temperature dependent properties are computed using the Monte Carlo

Metropolis method integrated with the adaptive move algorithm[8]. The dipole field contributions are computed using the macrocell approximation[9] with a 1 nm cell size and refined using a parallel fully atomistic dipole-dipole field calculation. A gyromagnetic ration of 1.76 $T^{-1}s^{-1}$ is used throughout the simulations. The atomistic calculation utilizes a recently implemented massively parallel algorithm allowing such calculations with full generality and high accuracy[10].

2 Simulation movies

Movie S1: Spin dynamics showing the cooling down process at zero magnetic field from 80 K down to 0 K for monolayer $CrCl_3$.

Movie S2: Similar as the Movie S1, but at a 50 mT magnetic field.

Movie S3: Similar as the Movie S1, but at a 500 mT magnetic field.

Movie S4: Spin dynamics at zero field and 0 K showing the collision process between a vortex and an antivortex since the early times when both are relatively isolated till they collapsed. Two antimerons are involved in this process.

Movie S5: Similar as Movie S4, but involving a meron and a antimeron.

Movie S6: Macro-dynamics of the magnetic domains showing the movement of the vortex and antivortex at the boundary between domain structures. The colors of the vortex and antivortex (blue and red) are kept the same as the one for the S^y component of the magnetization to simplify the analysis. However, the core of the spin textures are primarily composed by out-of-plane spins as highlighted in Figure 2.

Movie S7: Spin dynamics showing the cooling down process at zero magnetic field from 80 K down to 0 K for monolayer CrI_3.

Movie S8: Spin dynamics showing the cooling down process at zero magnetic field from 60 K down to 0 K for monolayer $CrBr_3$.

Movie S9: Spin dynamics showing the cooling down process at zero magnetic field from 80 K down to 0 K for monolayer CrF_3.

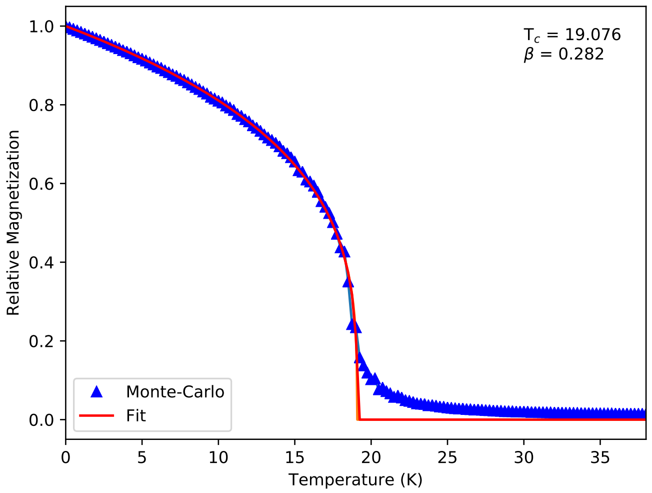

Figure S1: Calculated relative magnetization (a.u.) versus temperature (K) for monolayer $CrCl_3$ (blue triangles). Fit curve is obtained by using equation $M(T) = (1 - T/T_c)^{\beta}$, where T_c is the critical temperature and β the critical exponent. Converged values of T_c in Kelvin and β are included in the plot.

3 Calculation of the meron and anti-meron radius

The estimation of the full width at half maximum is done by fitting Pearson type VII function with m = 3 to our S^z data profile:

$$f(x) = I_{max}\left[1 + 4\left(2^{\frac{1}{3}} - 1\right)\left(\frac{x - x_0}{w_F}\right)^2\right]^{-3} \tag{1}$$

where I_{max} is the maximum value of the peak, x_0 is the center of the peak and w_F is the FWHM. The fitting immediately gives the FWHM but to extract the radius of the bubble, we have to find the x_{lim} s.t. $f(x < x_{lim}) < \alpha$, with α an arbitrary small value (we choose $\alpha = 10^{-2}$). Thus we need to solve the equation:

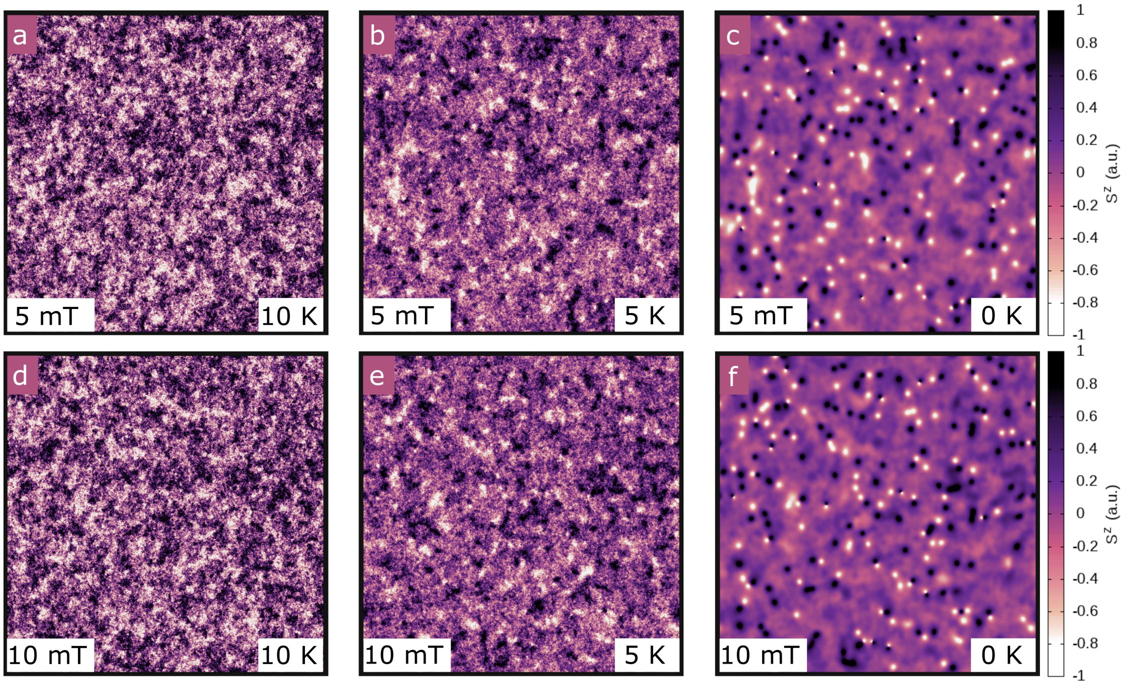

Figure S2: Spin dynamics of monolayer $CrCl_3$ at different magnitudes of magnetic field and temperatures: **a-c** 5 mT and **d-f** 10 mT. The out-of-plane spin component S^z is utilized to follow the nucleation of meron and antimerons across the surface (bright and dark dots).

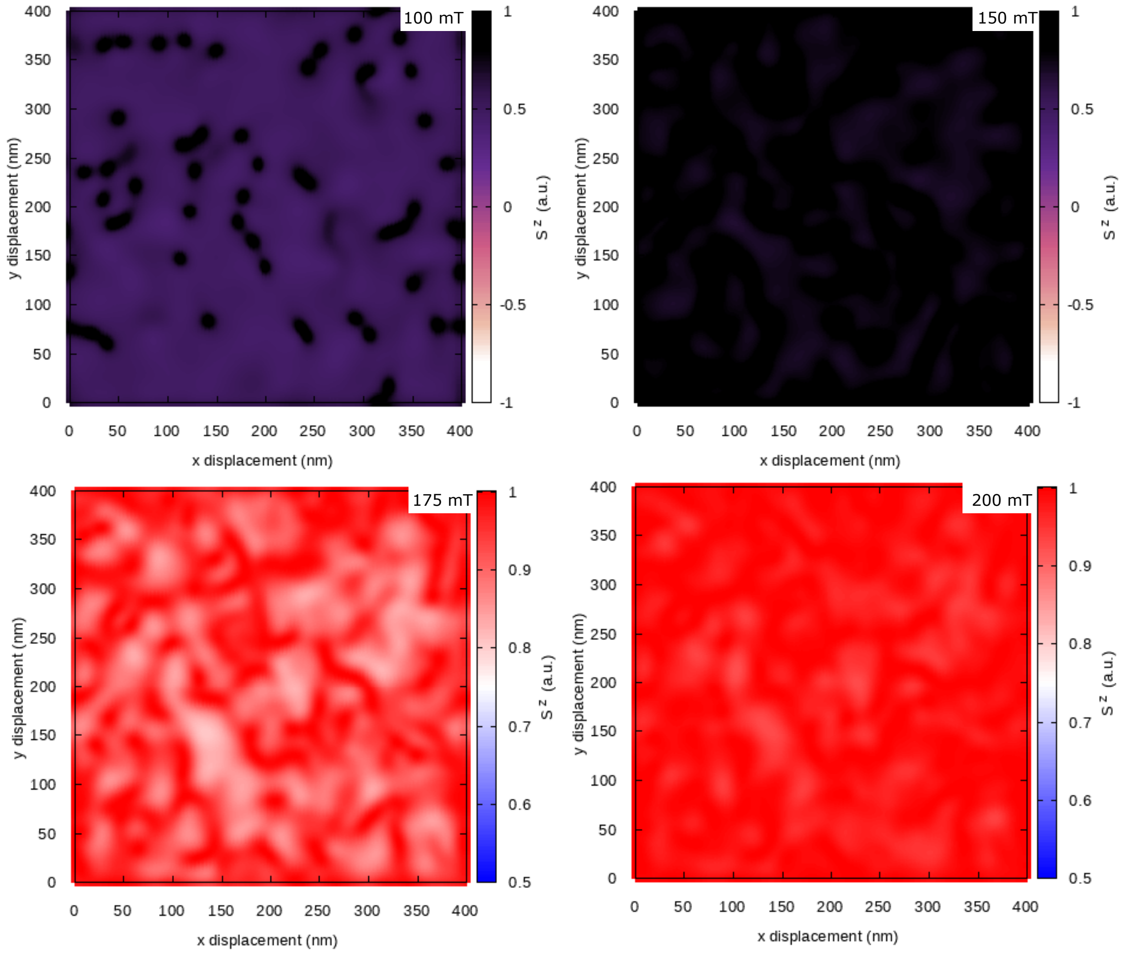

Figure S3: Spin dynamics of monolayer $CrCl_3$ at different magnitudes of magnetic field: 100 mT, 150 mT, 175 mT and 200 mT. The out-of-plane spin component S_z is utilized to follow the nucleation of meron and antimerons across the surface. At 100 mT, all nucleated spin textures have similar polarization ($S_z = 1$) with features following those at lower fields. For magnetic fields beyond 150 mT there is no formation of spin quasiparticles as all domains are strongly field polarized. A different scale colour (red-blue) is utilized for fields larger than 150 mT to allow observations of further features in the spin dynamics. Such colour scale is instrumental to identify small variations of S^z throughout the surface.

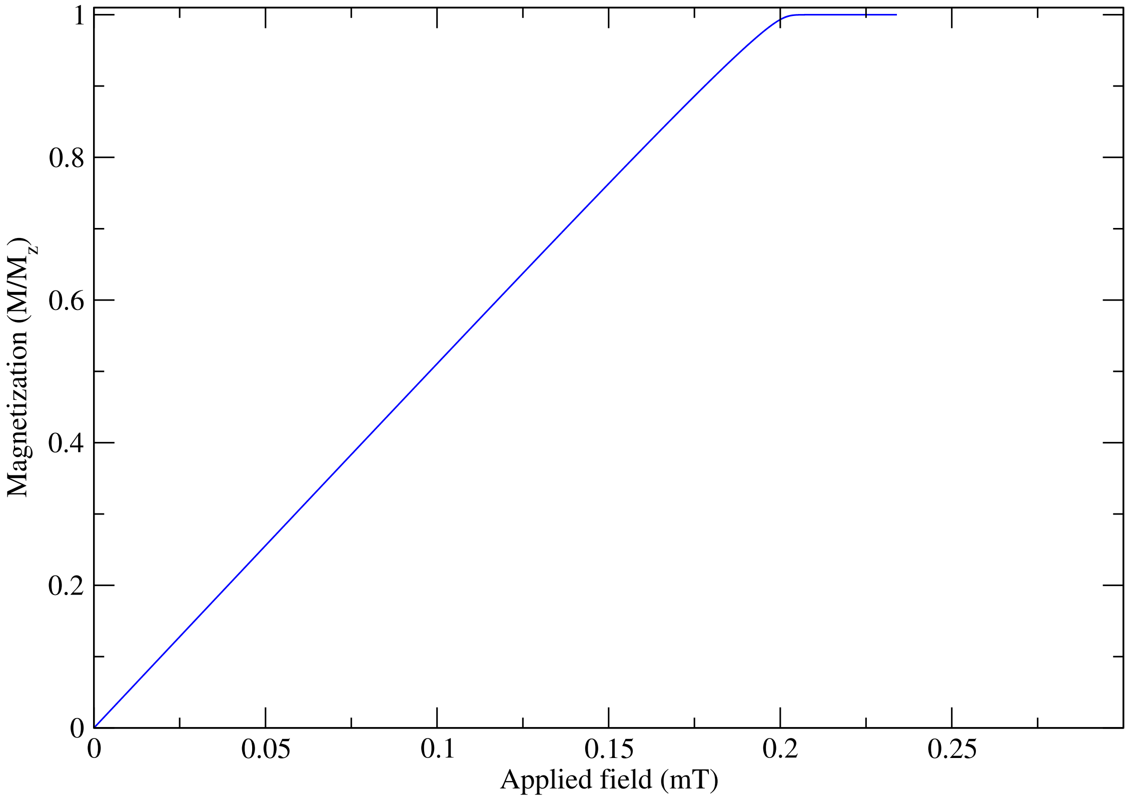

Figure S4: We estimated the critical magnetic field for monolayer $CrCl_3$ performing simulations on the hysteresis curve on one side of the full loop. The simulation were done with a starting field of 0 mT to a final 0.235 mT with an increment of 0.0005mT.

$$
\begin{aligned}
\alpha &= I_{max}\left[1+4\left(2^{\frac{1}{3}}-1\right)\left(\frac{x_{lim}-x_0}{w_F}\right)^2\right]^{-3} \\
\Rightarrow w_F^2+(x_{lim}-x_0)^2 &= \alpha^{-\frac{1}{3}} w_F^2 I_{max}^{\frac{1}{3}} \\
\Rightarrow 0 &= x_{lim}^2-2x_{lim}x_0+x_0^2+w_F^2\left(1-\alpha^{-\frac{1}{3}} I_{max}^{\frac{1}{3}}\right)
\end{aligned}
\tag{2}
$$

and thus we find:

$$
x_{lim}^{\pm} = x_0 \pm w_F\sqrt{\alpha^{-\frac{1}{3}} I_{max}^{\frac{1}{3}}-1} \tag{3}
$$

and

$$
R = \frac{x_{lim}^{+}-x_{lim}^{-}}{2} = w_F\sqrt{\alpha^{-\frac{1}{3}} I_{max}^{\frac{1}{3}}-1} \tag{4}
$$

4 Computing the topological quantum number

In the continuum case the topological charge Q is:

$$
Q = \frac{1}{4\pi}\int d^2r\left(\frac{\partial \boldsymbol{s}}{\partial x}\times\frac{\partial \boldsymbol{s}}{\partial y}\right)\cdot \boldsymbol{s} \tag{5}
$$

where $\boldsymbol{s}$ is the three-component spin field. This charge can also be defined for a lattice spin field $\boldsymbol{S}$:

$$
Q = \frac{1}{4\pi}\sum_{\Omega}\left[\Omega(\boldsymbol{S}_1,\boldsymbol{S}_2,\boldsymbol{S}_3)+\Omega(\boldsymbol{S}_1,\boldsymbol{S}_3,\boldsymbol{S}_4)\right] \tag{6}
$$

with $\Omega(\boldsymbol{S}_1,\boldsymbol{S}_2,\boldsymbol{S}_3)$ denoting the signed area of the spherical triangle with corner $\boldsymbol{S}_1,\boldsymbol{S}_2,\boldsymbol{S}_3$. The convention we used for the signed area is shown in Fig.S5 with the blue and red triangle.

The topological charge is obtained by gluing together all elementary triangles (the blue and red triangles in FigS5 are elementary triangles). The formula to compute the signed area Ω with unit spin vectors[1] is [12]:

$$\exp\left(i\frac{\Omega(\boldsymbol{S}_1,\boldsymbol{S}_2,\boldsymbol{S}_3)}{2}\right) = \rho^{-1}\left[1+\boldsymbol{S}_1\cdot\boldsymbol{S}_2+\boldsymbol{S}_2\cdot\boldsymbol{S}_3+\boldsymbol{S}_3\cdot\boldsymbol{S}_1+i\boldsymbol{S}_1\cdot(\boldsymbol{S}_2\times\boldsymbol{S}_3)\right] \tag{7}$$

where

$$\rho = \left[2\left(1+\boldsymbol{S}_1\cdot\boldsymbol{S}_2\right)\left(1+\boldsymbol{S}_2\cdot\boldsymbol{S}_3\right)\left(1+\boldsymbol{S}_3\cdot\boldsymbol{S}_1\right)\right]^{\frac{1}{2}}$$

the previous equation can be written as:

$$\tan\left(\frac{\Omega(\boldsymbol{S}_1,\boldsymbol{S}_2,\boldsymbol{S}_3)}{2}\right) = \frac{\boldsymbol{S}_1\cdot(\boldsymbol{S}_2\times\boldsymbol{S}_3)}{1+\boldsymbol{S}_1\cdot\boldsymbol{S}_2+\boldsymbol{S}_2\cdot\boldsymbol{S}_3+\boldsymbol{S}_3\cdot\boldsymbol{S}_1} \tag{8}$$

This kind of topological charge on honeycomb/hexagonal lattice was done in [13] and it gives the expected results for skyrmions.

To compute the topological charge of our bubbles we wrote a code:

- First we create a file containing only one bubble with the coordinate order in x. This gives a number of rows with the same x and different y coordinates as shown in Fig. S5 (the i, i+1, etc).

[1]We use this formula because vampire outputs unit vectors, one can find a more general formula in [11]

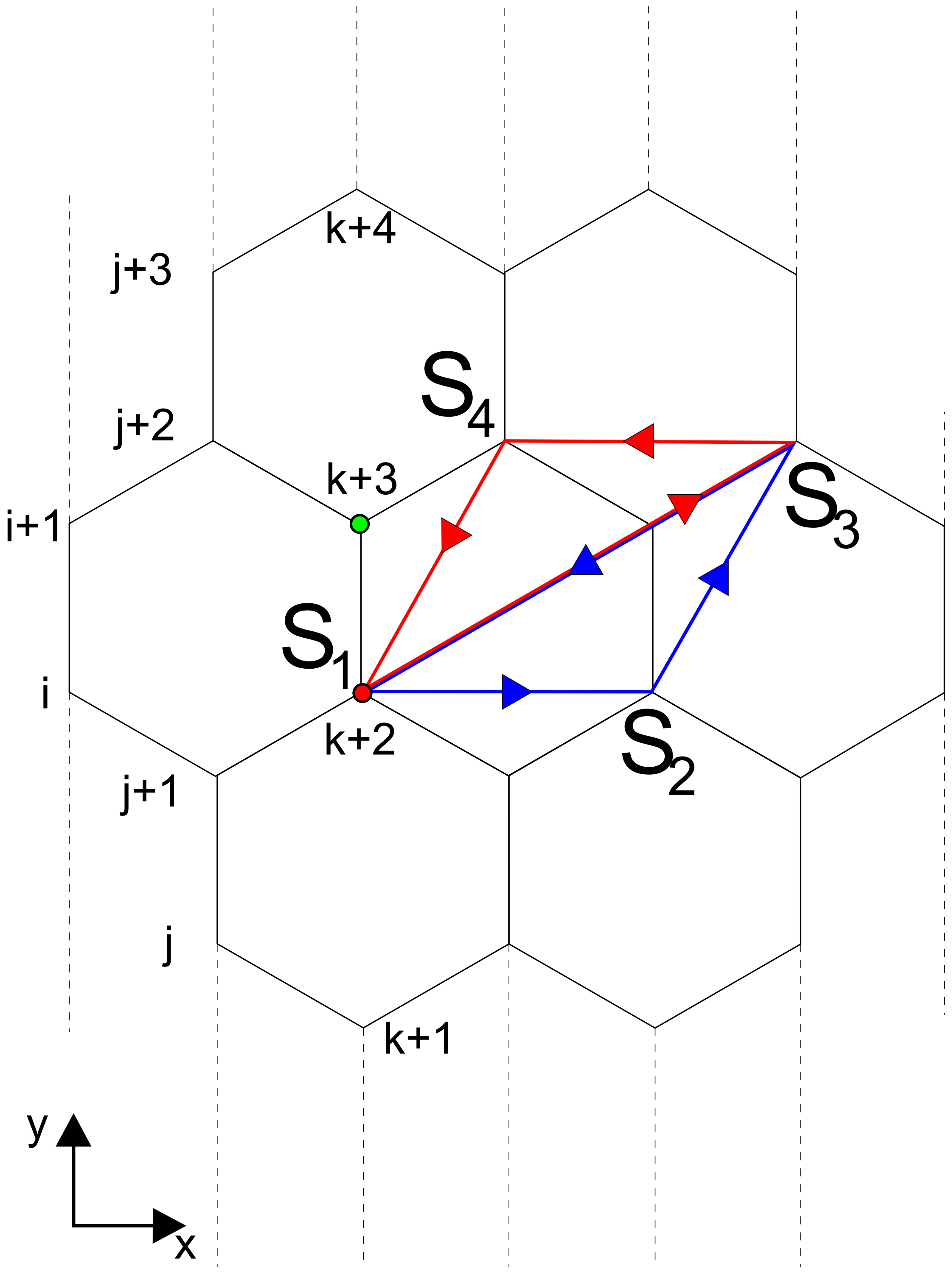

Figure S5: Honeycomb lattice showing spins S_i ($i = 1,2,3,4$) following a counter-clockwise order on each triangle grid. The different sites are denoted by j, k and i indexes.

- Then we choose a spin called $\boldsymbol{S}_1$ by taking the first row in the file (then the second, third,ith). From there we need to make sure to always keep the same convention for all triangles i.e., for a chosen $\boldsymbol{S}_1$ the associated $\boldsymbol{S}_2, \boldsymbol{S}_3, \boldsymbol{S}_4$ will always have the same relative position. Our convention is shown in Fig S5.

- To obtain $\boldsymbol{S}_2$ we check that the distance d between $\boldsymbol{S}_1$ and $\boldsymbol{S}_2$ is equal to the lattice parameter l_0 of $CrCl_3$[2], and we make sure that the y coordinate of both spins is the same. Because we look for $\boldsymbol{S}_2$ only in rows after the row of $\boldsymbol{S}_1$, our criteria make $\boldsymbol{S}_2$ unique.

- For $\boldsymbol{S}_4$ we use the criteria: $d\left(\boldsymbol{S}_1, \boldsymbol{S}_4\right) = l_0$, $d\left(\boldsymbol{S}_2, \boldsymbol{S}_4\right) = l_0$ and the y component of $\boldsymbol{S}_4$ is bigger than the y component of $\boldsymbol{S}_1$. Again, these criteria make $\boldsymbol{S}_4$ unique.

- Finally, for $\boldsymbol{S}_3$ we want $d\left(\boldsymbol{S}_2, \boldsymbol{S}_3\right) = l_0$, $d\left(\boldsymbol{S}_4, \boldsymbol{S}_3\right) = l_0$ and $d\left(\boldsymbol{S}_1, \boldsymbol{S}_3\right) = 12.26$ angstroms, making $\boldsymbol{S}_3$ unique.

5 Spin dynamics on other Cr-trihalide materials: CrX_3 (X=F, Br, I)

[2] $\boldsymbol{S}_1$ and $\boldsymbol{S}_2$ are second nearest neighbour in a honeycomb lattice

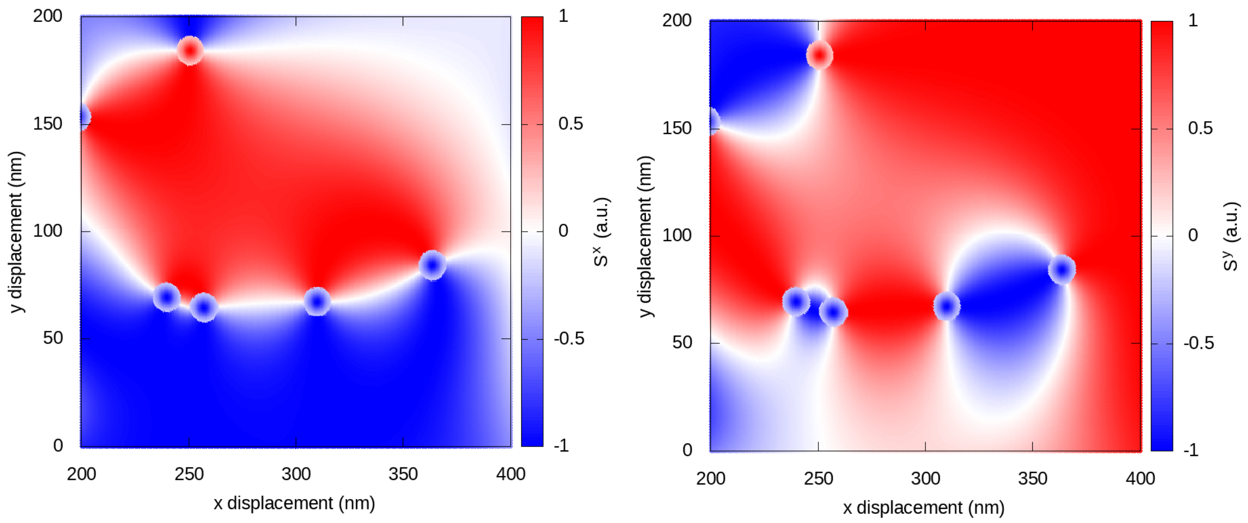

Figure S6: Snapshot of a magnetic domain configuration on monolayer $CrCl_3$ projected on in-plane spin components: S^x and S^y. The small circles (blue and red) show the different spin textures stabilized during the cooling process.

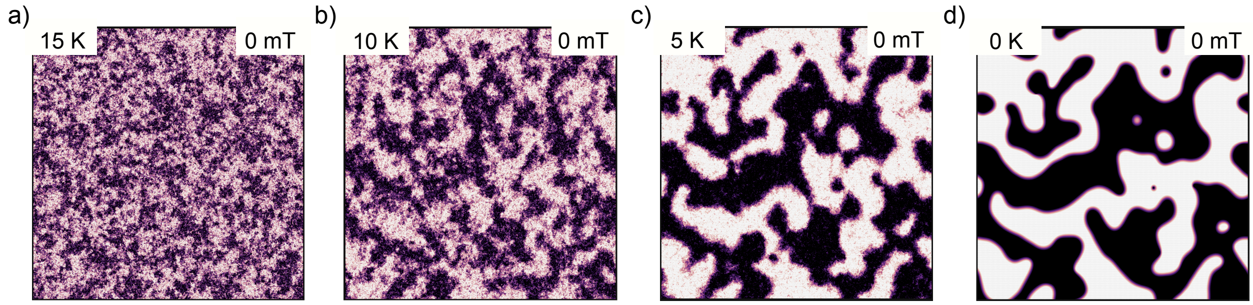

Figure S7: a)-d) Spin dynamics of monolayer $CrCl_3$ without taking into account dipole-dipole interactions at zero field and at different temperatures. As the system cools down, there is no appearance of topological spin-textures over the surface. The small bubbles observed in d) disappeared at longer times. Interestingly, the magnetic domains have out-of-plane magnetization following the single ion anisotropy. Only the S^z component is showed similarly as in Figure 1.

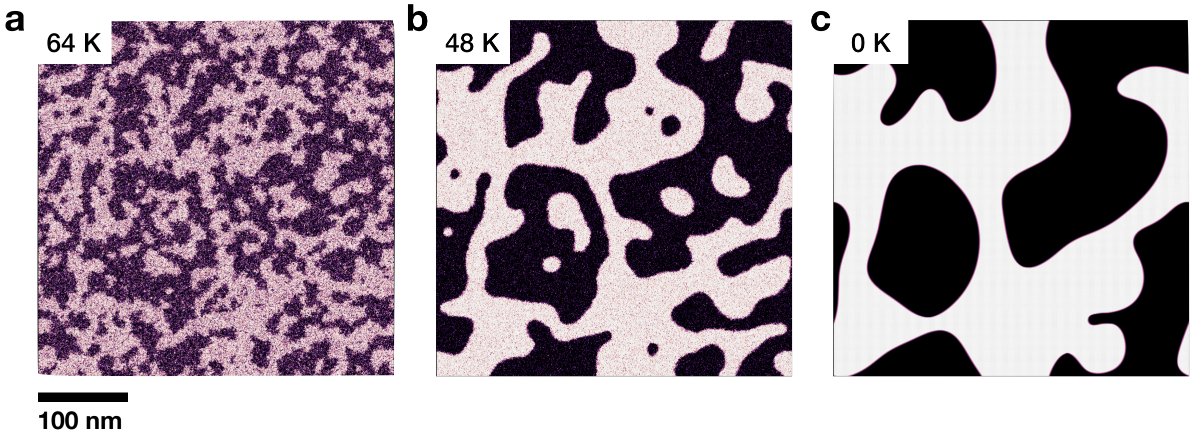

Figure S8: Zero-field cooling simulations for monolayer CrI_3 from temperatures above the Curie temperature (64 K) till 0 K. We use similar setup as those for monolayer $CrCl_3$. The out-of-plane component of the magnetization (S^z) is displayed in the plots. Dark/bright areas correspond to spins point either up or down to the surface.

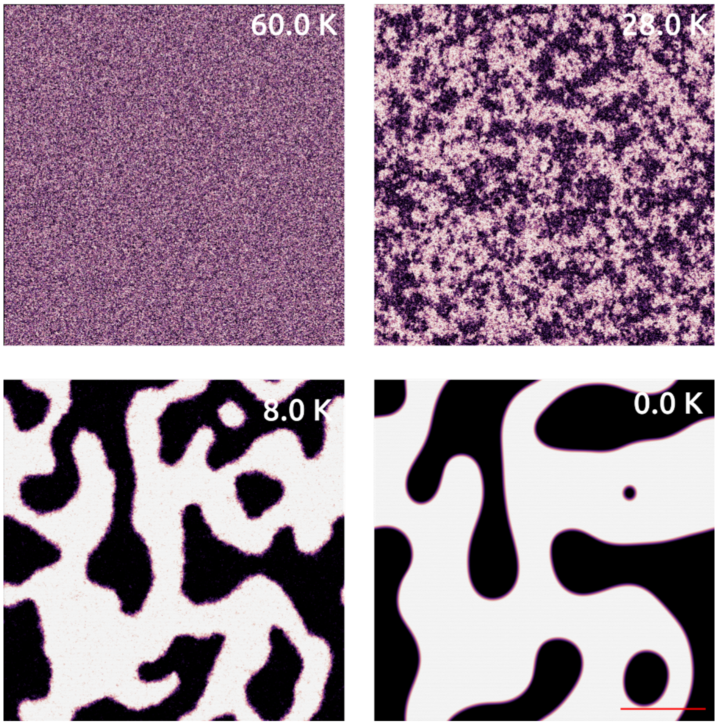

Figure S9: Zero-field cooling simulations for monolayer CrB_3 from temperatures above the Curie temperature (60 K) till 0 K. We use similar setup as those for monolayer $CrCl_3$. The out-of-plane component of the magnetization (S^z) is displayed in the plots. Dark/bright areas correspond to spins point either up or down to the surface. The scale bar is 100 nm.

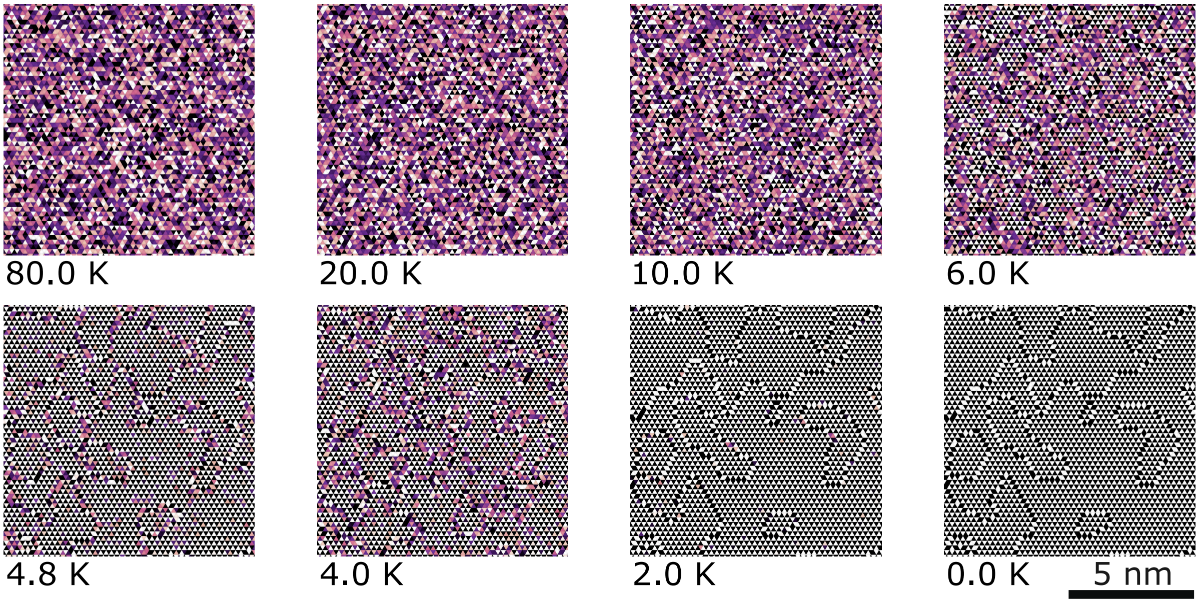

Figure S10: Zero-field cooling simulations for monolayer CrF_3 from temperatures above the Curie temperature (80 K) till 0 K. We use similar setup as those for monolayer $CrCl_3$. The out-of-plane component of the magnetization (S^z) is displayed in the plots. Dark/bright areas correspond to spins point either up or down to the surface. Due to the anti-ferromagnetic character of the exchange coupling in CrF_3 the magnetic domains look differently than thee other Cr-trihalides.

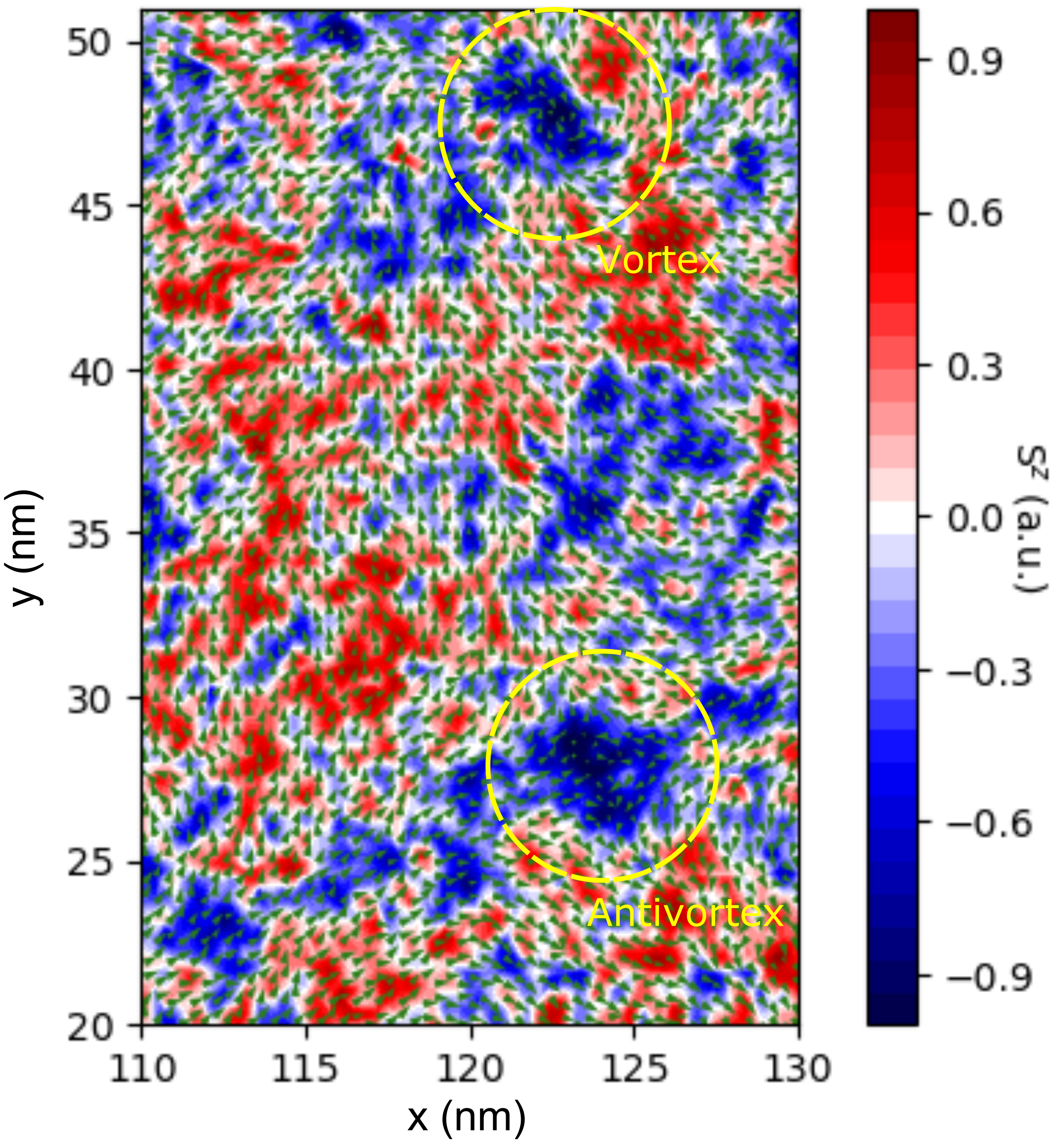

Figure S11: Snapshot of a spin dynamics of $CrCl_3$ at 8 K without considering dipolar interactions at any direction but setting an easy-plane (XY) for the magnetic anisotropy[14,15].

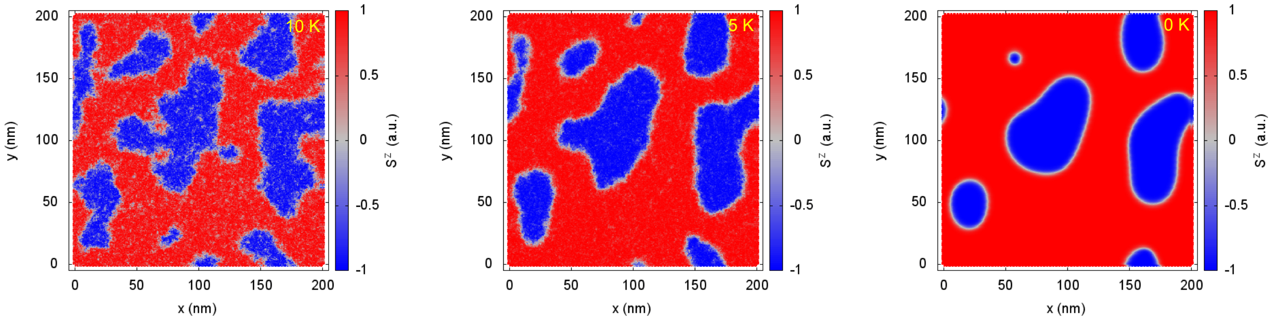

Figure S12: Snapshot of a spin dynamics of $CrCl_3$ at different temperatures (10 K, 5 K, 0 K) without considering second- and third-nearest neighbours (J_{2nd}, J_{3rd}, λ_{2nd}, λ_{3rd}) but including dipolar interactions and biquadratic exchange into the simulations. There is no formation of vortex or antivortex spin textures throughout the system at this level of theory.

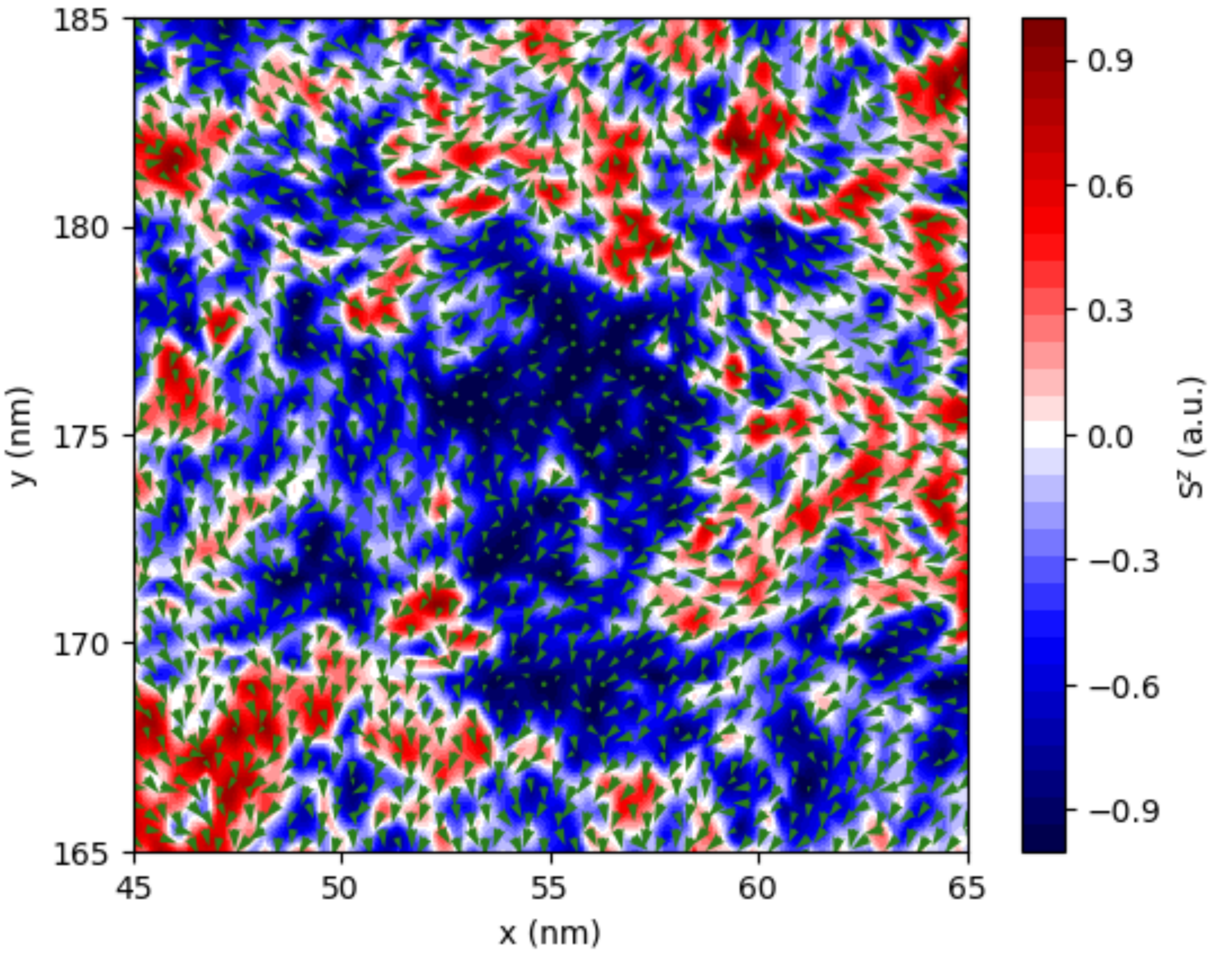

Figure S13: Snapshot of a spin dynamics of $CrCl_3$ at 8 K without considering biquadratic exchange but including up to third-nearest neighbours on bilinear exchange and dipolar interactions. The formation of merons and antimerons become more chaotic on the spin patterns.

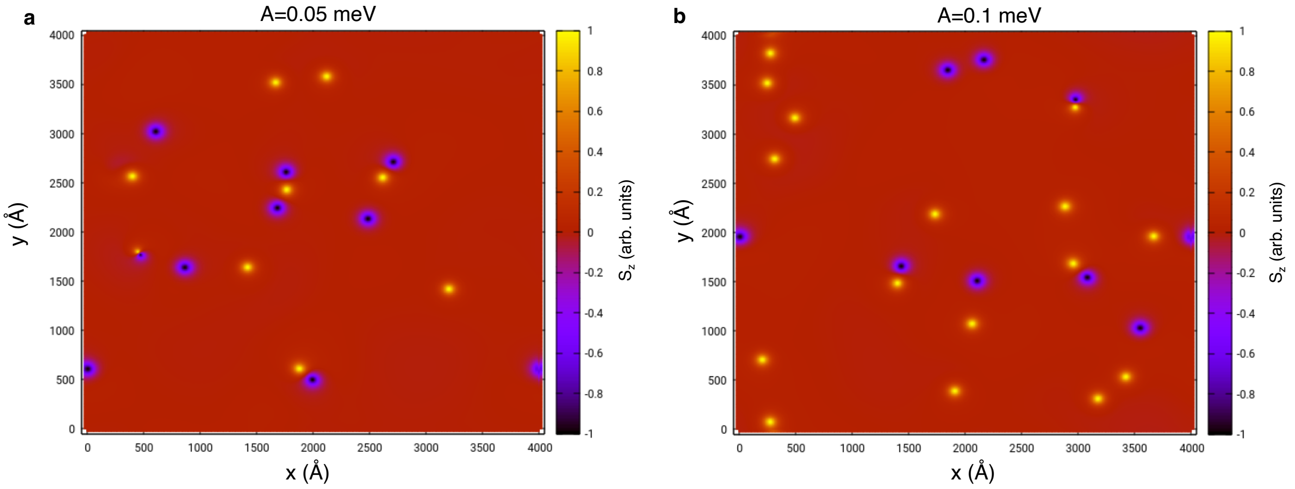

Figure S14: **a-b,** Snapshots of a spin dynamics of $CrCl_3$ at 0 K taking into account Dzyaloshinskii-Moriya interaction (DMI) with $A = 0.05$ mev and $A = 0.1$ meV, respectively. DMI is included in Eq. 1 via an additional term given by $A\sum_{i,j}(S_i \times S_j)$, where A is the strength of DMI and $S_{i,j}$ the spin vectors. We considered DMI perpendicular to the surface at second nearest neighbours since there is none at the first-nearest neighbours due to the symmetry of the lattice. The out-of-plane component of the magnetization S^z is utilized for following the evolution of the spin-textures. We have observed no modifications of the dynamics of merons and antimerons in monolayer $CrCl_3$ with the inclusion of DMI.

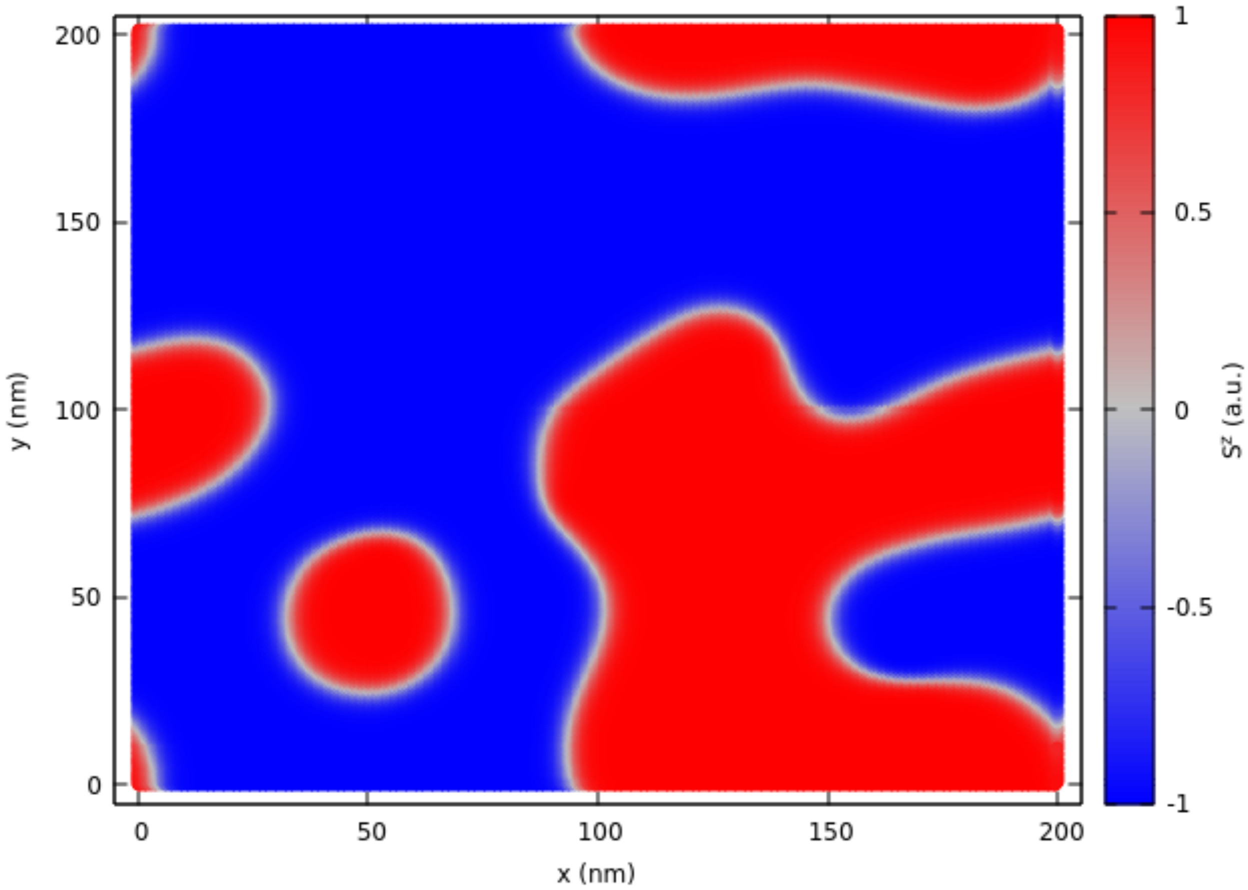

Figure S15: Snapshot of a spin dynamics of $CrCl_3$ at 0 K considering a single-ion anisotropy slightly larger (36 μeV) than that considered in the other simulations (12.67 μeV) in this study. The fluctuations are still present but now with all spins pointing out-of-plane along S^z with no formation of merons or antimerons.

References

1. Kresse, G. & Furthmüller, J. Efficient iterative schemes for ab initio total-energy calculations using a plane-wave basis set. *Physical review B* **54**, 11169 (1996).

2. Perdew, J. P., Burke, K. & Ernzerhof, M. Generalized gradient approximation made simple. *Phys. Rev. Lett.* **77**, 3865–3868 (1996). URL https://link.aps.org/doi/10.1103/PhysRevLett.77.3865.

3. Dudarev, S. L., Botton, G. A., Savrasov, S. Y., Humphreys, C. J. & Sutton, A. P. Electron-energy-loss spectra and the structural stability of nickel oxide: An lsda+u study. *Phys. Rev. B* **57**, 1505–1509 (1998). URL https://link.aps.org/doi/10.1103/PhysRevB.57.1505.

4. Liu, J., Sun, Q., Kawazoe, Y. & Jena, P. Exfoliating biocompatible ferromagnetic cr-trihalide monolayers. *Physical Chemistry Chemical Physics* **18**, 8777–8784 (2016).

5. Kartsev, A., Augustin, M., Evans, R. F. L., Novoselov, K. S. & Santos, E. J. G. Higher-order exchange interactions in two-dimensional magnets. *arXiv:2006.04891* (2020).

6. Evans, R. F. L. *et al.* Atomistic spin model simulations of magnetic nanomaterials. *Journal of Physics: Condensed Matter* **26**, 103202 (2014). URL https://doi.org/10.1088%2F0953-8984%2F26%2F10%2F103202.

7. Ellis, M. O. A. *et al.* The landau–lifshitz equation in atomistic models. *Low Temperature Physics* **41**, 705–712 (2015). URL https://doi.org/10.1063/1.4930971. https://doi.org/10.1063/1.4930971.

8. Alzate-Cardona, J. D., Sabogal-Suárez, D., Evans, R. F. L. & Restrepo-Parra, E. Optimal phase space sampling for monte carlo simulations of heisenberg spin systems. *Journal of Physics: Condensed Matter* **31**, 095802 (2019). URL https://doi.org/10.1088%2F1361-648x%2Faaf852.

9. Bowden, G. J., Stenning, G. B. G. & van der Laan, G. Inter and intra macro-cell model for point dipole-dipole energy calculations. *Journal of Physics: Condensed Matter* **28**, 066001 (2016). URL http://stacks.iop.org/0953-8984/28/i=6/a=066001.

10. VAMPIRE software package. Version 6. Available from http://vampire.york.ac.uk/.

11. Van Oosterom, A. & Strackee, J. The solid angle of a plane triangle. *IEEE Transactions on Biomedical Engineering* **BME-30**, 125–126 (1983).

12. Berg, B. & Lüscher, M. Definition and statistical distributions of a topological number in the lattice o(3) -model. *Nuclear Physics B* **190**, 412 – 424 (1981). URL http://www.sciencedirect.com/science/article/pii/055032138190568X.

13. Rózsa, L., Simon, E., Palotás, K., Udvardi, L. & Szunyogh, L. Complex magnetic phase diagram and skyrmion lifetime in an ultrathin film from atomistic simulations. *Phys. Rev. B* **93**, 024417 (2016). URL https://link.aps.org/doi/10.1103/PhysRevB.93.024417.

14. Vedmedenko, E. Y., Oepen, H. P., Ghazali, A., Lévy, J.-C. S. & Kirschner, J. Magnetic microstructure of the spin reorientation transition: A computer experiment. *Phys. Rev. Lett.* **84**, 5884–5887 (2000). URL https://link.aps.org/doi/10.1103/PhysRevLett.84.5884.

15. Gouva, M. E., Wysin, G. M., Bishop, A. R. & Mertens, F. G. Vortices in the classical two-dimensional anisotropic heisenberg model. *Phys. Rev. B* **39**, 11840–11849 (1989). URL https://link.aps.org/doi/10.1103/PhysRevB.39.11840.
