# Supplementary material for: Properties and dynamics of meron topological spin textures in the two-dimensional magnet CrCl3
Source: Nat Commun. 2021 Jan 8;12:185. doi: 10.1038/s41467-020-20497-2 (PMC7794561; doi:10.1038/s41467-020-20497-2)
Supplement: Supplementary file 3 — Description of Additional Supplementary Files [file 41467_2020_20497_MOESM3_ESM.pdf]

Title: Supplementary Movie 1

Description: Spin dynamics showing the cooling down process at zero magnetic fiel from 80 K down to 0 K for monolayer CrCl3.

Title: Supplementary Movie 2

Description: Similar as the Movie S1, but at a 50 mT magnetic field.

Title: Supplementary Movie 3

Description: Similar as the Movie S1, but at a 500 mT magnetic field.

Title: Supplementary Movie 4

Description: Spin dynamics at zero field and 0 K showing the collision process between a vortex and an antivortex since the early times when both are relatively isolated till they collapsed. Two antimerons are involved in this process.

Title: Supplementary Movie 5

Description: Similar as Movie S4, but involving a meron and a antimeron.

Title: Supplementary Movie 6

Description: Macro-dynamics of the magnetic domains showing the movement of the vortex and antivortex at the boundary between domain structures. The colors of the vortex and antivortex (blue and red) are kept the same as the one for the Sy component of the magnetization to simplify the analysis. However, the core of the spin textures are primarily composed by out-of-plane spins as highlighted in Figure 2.

Title: Supplementary Movie 7

Description: Spin dynamics showing the cooling down process at zero magnetic fiel from 80 K down to 0 K for monolayer CrI3.

Title: Supplementary Movie 8

Description: Spin dynamics showing the cooling down process at zero magnetic field from 60 K down to 0 K for monolayer CrBr3.

Title: Supplementary Movie 9

Description: Spin dynamics showing the cooling down process at zero magnetic fiel from 80 K down to 0 K for monolayer CrF3.
